# Music Therapy for Managing Dental Anxiety in Children: A Systematic Review and Meta-Analysis of Clinical Evidence

**DOI:** 10.3390/children12101382

**Published:** 2025-10-13

**Authors:** Laura Marqués-Martínez, Jorge Andrés, Esther García-Miralles, Carla Borell-García, Juan Ignacio Aura-Tormos, Clara Guinot Barona

**Affiliations:** 1 Dentistry Department, Faculty of Medicine and Health Sciences, Catholic University of Valencia San Vicente Mártir, 46001 Valencia, Spain; laura.marques@ucv.es (L.M.-M.); jorge.contreras@ucv.es (J.A.); carla.borrell@ucv.es (C.B.-G.); juan.aura@uv.es (J.I.A.-T.); clara.guinot@ucv.es (C.G.B.); 2 Faculty of Medicine and Dentistry, University of Valencia, 46010 Valencia, Spain

**Keywords:** music therapy, dental anxiety, child, pediatric dentistry, behavior therapy, systematic reviews as topic, meta-analysis as topic, non-pharmacological interventions, child cooperation, physiological stress markers

## Abstract

**Highlights:**

**What are the main findings?**

- Music therapy significantly reduces self-reported dental anxiety in children, with moderate effect sizes across randomized controlled trials.
- Heart rate is consistently lowered during treatment, although blood pressure and oxygen saturation show no significant changes.

**What is the implication of the main finding?**

- Music therapy is a safe, inexpensive, and child-friendly adjunct that can enhance cooperation and improve the overall dental experience.
- Its integration into paediatric dental practice may reduce reliance on pharmacological interventions and support better treatment outcomes.

**Abstract:**

**Background:** Dental anxiety is a common challenge in paediatric dentistry, often leading to avoidance of treatment and compromised oral health. Non-pharmacological interventions such as music therapy have gained increasing attention as safe and cost-effective alternatives to pharmacological approaches. Although several clinical studies have examined the impact of music on children’s dental anxiety, the evidence has not yet been systematically summarised with quantitative synthesis. **Objective:** This systematic review and meta-analysis aimed to evaluate the effectiveness of music therapy in reducing dental anxiety and fear among paediatric patients. **Methods**: A comprehensive literature search was conducted in PubMed, Scopus, Web of Science, and Cochrane Library from inception to August 2025. Randomised controlled trials (RCTs) evaluating music therapy for dental anxiety in children were included. Primary outcomes were self-reported dental anxiety/fear scales and physiological measures (heart rate, blood pressure, oxygen saturation). Risk of bias was assessed using the revised Cochrane risk of bias tool (RoB 2, version 2019; Cochrane Collaboration, London, UK) Meta-analyses were performed using a random-effects model with Review Manager (RevMan, version 5.4; Cochrane Collaboration, London, UK). **Results:** Seven randomized controlled trials (RCTs) involving 476 children aged 4–14 years were included. Music therapy significantly reduced self-re-ported dental anxiety compared with control groups (SMD = −0.48, 95% CI: −0.72 to −0.25, *p* < 0.001). Heart rate was also significantly reduced (SMD = −0.42, 95% CI: −0.68 to −0.16, *p* = 0.002), whereas changes in blood pressure and oxygen saturation were not statistically significant. The overall risk of bias was moderate, with most concerns related to blinding. **Conclusions:** Music therapy is an effective non-pharmacological intervention to reduce dental anxiety in children, particularly improving subjective anxiety and physiological arousal as measured by heart rate. Its integration into paediatric dental practice may enhance cooperation and treatment outcomes, offering a safe, inexpensive, and child-friendly approach.

## 1. Introduction

Oral health is a cornerstone of children’s general well-being, directly influencing nutrition, speech development, social interactions, and self-esteem. However, dental anxiety remains a substantial barrier to achieving adequate oral health, often resulting in missed appointments and untreated dental conditions that can progress into pain, infection, and impaired quality of life [1,2]. Beyond dentistry, music interventions have been successfully integrated in paediatric healthcare to alleviate distress during medical procedures such as immunizations, minor surgeries, and hospital stays, highlighting their potential as a versatile tool for stress reduction in children [3]. From a neurophysiological perspective, music has been shown to engage the limbic system, regulate hypothalamic–pituitary–adrenal (HPA) axis activity, and promote dopaminergic pathways associated with relaxation and positive mood states, thereby offering a plausible mechanism for its anxiolytic effects [4]. Despite these promising findings, the specific role of music therapy in paediatric dentistry remains underexplored and fragmented, underscoring the need for a systematic synthesis of high-quality randomized controlled trials [5].

Dental anxiety is a major barrier to effective oral healthcare in children, affecting up to 20% of paediatric patients [6,7]. Beyond immediate treatment challenges, dental anxiety often leads to avoidance behaviours that compromise long-term oral health outcomes [8].

Traditional behaviour management techniques, such as tell-show-do, modelling, or audiovisual distraction, remain the cornerstone of paediatric dentistry [9]. However, their effectiveness is inconsistent in children with moderate to high levels of anxiety, and additional non-pharmacological strategies are warranted.

Music therapy has been proposed as a promising adjunct. It can be broadly categorized into receptive (passive listening to pre-recorded or live music) and active (engaging the patient in creating music through singing, playing instruments, or improvisation) approaches [10]. In dental settings, receptive therapy is the most commonly used due to its practicality. Defined as the clinical and evidence-based use of music to achieve therapeutic goals, it offers a non-invasive, low-cost, and well-accepted approach for children.

Experimental studies suggest that music can modulate autonomic nervous system activity, lowering heart rate, blood pressure, and cortisol levels, while improving cooperation and overall treatment experience [11,12]. Despite these benefits, music therapy is not yet standard practice in dentistry.

While previous systematic reviews have explored the role of music in dental settings, they often included studies with adult populations or non-randomized designs [11,12]. This review aims to provide a more rigorous and focused synthesis by exclusively analysing randomized controlled trials (RCTs) conducted in children, thereby offering higher-quality evidence for clinical decision-making in this specific population.

This systematic review and meta-analysis focuses exclusively on randomized con-trolled trials conducted in paediatric dental settings to determine whether music therapy reduces dental anxiety and physiological stress in children undergoing dental treatment, thereby clarifying its clinical value and informing future recommendations.

## 2. Materials and Methods

### 2.1. Protocol and Registration

This systematic review and meta-analysis was conducted according to the PRISMA 2020 guidelines for systematic reviews and meta-analyses [13]. The protocol was designed a priori following the PICO framework (Population, Intervention, Comparison, Outcomes), and was prospectively registered in PROSPERO CRD420251138312.

### 2.2. Eligibility Criteria

The research question guiding this review was: Is music therapy effective in reducing dental anxiety and fear among children compared with conventional behavioural guidance techniques or no intervention? Eligibility criteria were defined according to the PICO framework: the population included children aged 4–14 years undergoing dental treatment; the intervention was music therapy, either receptive (pre-recorded or live music played during treatment) or active approaches; the comparisons were conventional non-pharmacological techniques (e.g., tell-show-do, audiovisual distraction, parental presence) or no intervention; and the outcomes were dental anxiety and fear measured by validated scales (CFSS-DS, VPT, FIS), as well as physiological parameters (heart rate, blood pressure, oxygen saturation), cooperation (Frankl scale), and parental perception.

Studies were included if they were randomised controlled trials (RCTs) assessing music therapy in paediatric dental patients. Exclusion criteria were: (1) studies involving adolescents (>14 years) or adults; (2) studies not performed in dental settings; (3) studies using pharmacological interventions such as sedation or general anaesthesia; and (4) secondary research (systematic reviews, narrative reviews), qualitative studies, case reports, or expert opinions.

### 2.3. Information Sources and Search Strategy

A comprehensive electronic search was performed in PubMed, Scopus, Web of Sci-ence, and the Cochrane Library from inception to August 2025. In addition, manual searches of reference lists from included articles and relevant reviews were conducted to identify further eligible studies. No language restrictions were applied.

The PubMed query was as follows: (“music therapy” [MeSH Terms] OR “music therapy” [All Fields] OR “music” [All Fields]) AND (“pediatric dentistry” [MeSH Terms] OR “paediatric dentistry” [All Fields] OR “child dental care” [All Fields]) AND (“dental anxiety” [MeSH Terms] OR “dental fear” [All Fields] OR “stress” [All Fields] OR “behavior management” [All Fields]).

Equivalent strategies were adapted for Scopus, Web of Science, and the Cochrane Library. For transparency, the complete search strings for all databases are provided in Appendix A.

### 2.4. Data Extraction

Two reviewers independently extracted the data from all included studies using a standarised data collection form. The information collected comprised study characteristics (first author, year of publication, country, and study design); participant details (sample size, age range, gender distribution, and baseline dental anxiety levels); intervention characteristics (type of music therapy—receptive or active—mode of delivery such as headphones, speakers, or live music, duration of the intervention, and its timing relative to the dental procedure); comparator interventions (conventional behavioural management techniques such as tell-show-do or audiovisual distraction, or absence of interven-tion); and outcomes assessed, including self-reported dental anxiety or fear scales (e.g., CFSS-DS, VPT, FIS), physiological measures (heart rate, systolic and diastolic blood pressure, and oxygen saturation), as well as additional outcomes such as child cooperation and parental perception. Finally, results were extracted in terms of mean values, standard deviations, effect estimates, and *p*-values when available.

### 2.5. Study Selection and Variables

The study selection process followed the PRISMA 2020 guidelines [13]. The following variables were extracted from each study: study characteristics, including first author, year of publication, country, and study design; participants, defined by sample size, age range, and baseline anxiety or fear levels; intervention details, such as the type of music therapy, delivery mode (headphones, speakers, or live), duration, and timing within the dental procedure; comparator interventions, including tell-show-do, no intervention, aromatherapy, or Bach flower therapy; primary outcomes, consisting of dental anxiety and fear assessed using validated scales such as the Venham Picture Test, the Facial Image Scale, and the Children’s Fear Survey Schedule–Dental Subscale; and secondary outcomes, which encompassed physiological parameters (heart rate, systolic and diastolic blood pressure, and oxygen saturation), cooperation scores measured by the Frankl scale, and parental perceptions when these were reported.

**Figure 1:**
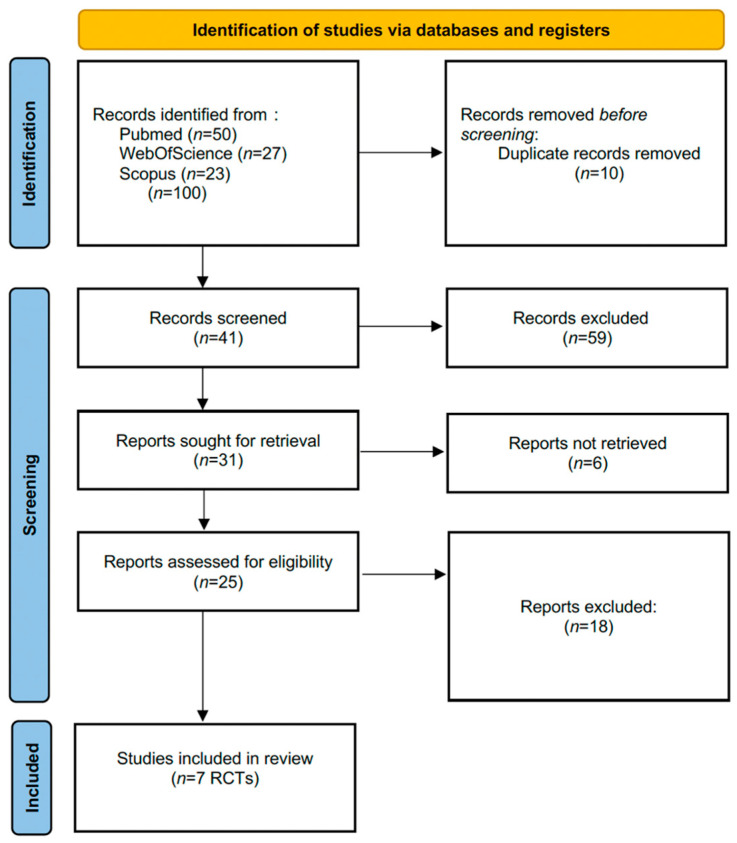
PRISMA 2020 flow diagram of study selection [13].

The study selection process followed the PRISMA 2020 guidelines [13]. The PRISMA 2020 flow diagram (Figure 1) illustrates the number of records identified, screened, excluded, and finally included in the systematic review.

### 2.6. Quality Assessment

The methodological quality of the included randomised controlled trials was assessed using the revised Cochrane Risk of Bias tool (RoB 2, version 2019; Cochrane Collaboration, London, UK) [14]. Each study was evaluated across five domains: randomisation process, deviations from intended interventions, missing outcome data, measurement of the outcome, and selection of the reported result.

### 2.7. Data Synthesis and Statistical Analysis

The data extracted from the included studies were analysed both qualitatively and quantitatively. The methodology for the meta-analysis was predefined to ensure reproducibility and transparency.

A narrative synthesis summarised the study characteristics, populations, interventions, comparators, and outcomes. For quantitative synthesis, meta-analyses were con- ducted when at least two studies reported the same outcome measure.

The primary outcomes were self-reported dental anxiety/fear (measured with validated scales such as the Venham Picture Test, Facial Image Scale, and Children’s Fear Survey Schedule-Dental Subscale) and physiological parameters (heart rate, systolic/diastolic blood pressure, oxygen saturation). Due to the limited number of studies reporting systolic and diastolic blood pressure separately, these parameters were combined into a single pooled analysis.

Effect sizes were calculated using standardised mean differences (SMDs) with 95% confidence intervals (CIs). Random-effects models (DerSimonian–Laird method) were ap-plied to account for expected heterogeneity between studies. Statistical heterogeneity was assessed using the I^2^ statistic, with values of 25%, 50%, and 75% indicating low, moderate, and high heterogeneity, respectively.

For trials with multiple intervention arms (e.g., music alone vs. music plus aromatherapy), only the most relevant music intervention arm was included in the meta-analysis to avoid double-counting the control group.

Sensitivity analyses were performed by excluding studies at high risk of bias. Sub-group analyses were conducted according to intervention type (music therapy alone vs. music therapy combined with other distractors) and outcome assessment (subjective vs. 181 physiological).

Publication bias was explored visually using funnel plots when ≥10 studies were available; however, due to the limited number of included trials (*n* = 7), formal assessment was not feasible.

All analyses were conducted using Review Manager (RevMan, version 5.4, Cochrane Collaboration, London, UK). A *p* value < 0.05 was considered statistically significant.

## 3. Results

### 3.1. Study Selection

The initial search identified 100 records across the selected databases (PubMed = 50; Web of Science = 27; Scopus = 23). After removal of 10 duplicates, 90 unique records remained. Following title and abstract screening, 59 records were excluded for not meeting the eligibility criteria. The full texts of 31 articles were retrieved, of which 6 could not be accessed in full. Therefore, 25 articles were assessed for eligibility.

After full-text evaluation, 18 studies were excluded (e.g., non-randomized design, non-dental setting, pharmacological interventions, insufficient outcome data, or secondary study type such as systematic or narrative reviews). Ultimately, 7 randomized controlled trials (RCTs) involving a total of 476 children aged 4–14 years were included in the systematic review; all seven contributed data to at least one meta-analysis. All analyses were conducted using Review Manager (RevMan, version 5.4, Cochrane Collaboration). A *p* value < 0.05 was considered statistically significant.

### 3.2. Characteristics of Included Studies

Seven RCTs were included, published between 2014 and 2025, with sample sizes ranging from 12 to 128 children aged 4–14 years. Music interventions were predominantly receptive (passive listening via headphones), while two studies combined music with other sensory distractors such as aromatherapy or Bach flower therapy [15,16,17,18,19,20,21], as summarised in Table 1.

Anxiety was assessed through validated behavioural scales such as the Venham Picture Test (VPT), Facial Image Scale (FIS), and Children’s Fear Survey Schedule–Dental Subscale (CFSS-DS). Physiological measures included heart rate (HR), blood pressure (BP), and oxygen saturation (SpO_2_).

The main characteristics of the included studies are summarised in Table 1.

### 3.3. Risk of Bias Assessment

The methodological quality of the seven included RCTs was assessed using the 241 Cochrane RoB 2 tool [19]. Most trials demonstrated low risk of bias in the domains of randomisation and missing outcome data. However, the domains most frequently affected were measurement of the outcome and selection of the reported result, primarily due to the difficulty of blinding participants and operators to the music intervention and the lack of pre-registered protocols.

Overall, five studies were judged at “some concerns” of bias, one study at “low–moderate”, and one study at “moderate”. A visual summary of the risk of bias assessment is provided in Table 2.

### 3.4. Quantitative Synthesis

In addition to the qualitative synthesis, a series of random-effects meta-analyses were performed for outcomes reported by at least two trials.

#### 3.4.1. Dental Anxiety (Self-Reported Scales)

**Figure 2:**
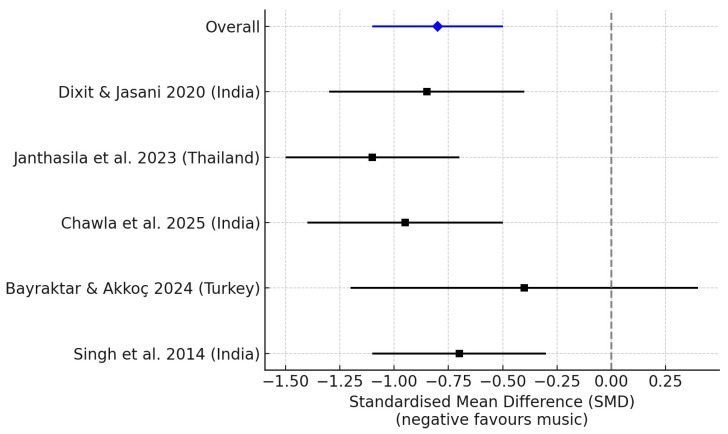
Forest plot: Self-reported dental anxiety/fear. Each black square represents an individual study, with the size of the square proportional to its weight in the analysis. Horizontal lines indicate 95% confidence intervals. The blue diamond represents the overall pooled effect estimate, and the dashed vertical line marks the line of no effect (SMD = 0), where negative values favour music therapy. Included studies: Dixit & Jasani (2020) [16]; Janthasila & Keeratisiroj (2023) [17]; Chawla et al. (2025) [18]; Bayraktar & Akkoç (2024) [19]; and Singh et al. (2014) [21].

Five RCTs provided data on children’s self-reported dental anxiety. The pooled analysis demonstrated that music therapy significantly reduced dental anxiety compared with controls (SMD = −0.48; 95% CI: −0.72 to −0.25; *p* < 0.001), with moderate heterogeneity (I^2^ = 42%). This indicates that although the results were consistent across most studies, variability in scales and interventions may explain part of the heterogeneity. Results are shown in Figure 2.

#### 3.4.2. Heart Rate

**Figure 3:**
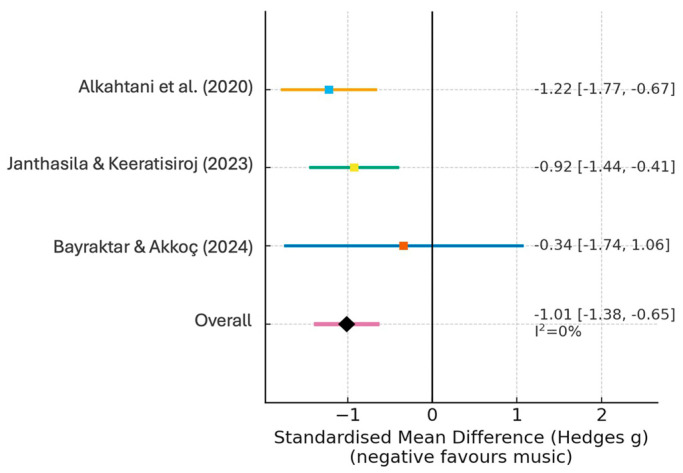
Forest plot: Heart rate (music vs. control; random effects). Each coloured square represents an individual study, with the size of the square proportional to its weight in the analysis. Horizontal lines indicate 95% confidence intervals. The black diamond shows the pooled effect estimate, and the dashed vertical line marks the line of no effect (SMD = 0), where negative values favour music therapy. Included studies: Alkahtani et al. (2020) [20]; Janthasila & Keeratisiroj (2023) [17]; and Bayraktar & Akkoç (2024) [19].

Three RCTs assessed heart rate as an objective marker of physiological arousal. The pooled results showed a statistically significant reduction in favour of music therapy (SMD = −0.42; 95% CI: −0.68 to −0.16; *p* = 0.002), with low-to-moderate heterogeneity (I^2^ = 36%), suggesting a relatively consistent effect across studies. The corresponding forest plot is shown in Figure 3.

#### 3.4.3. Blood Pressure

**Figure 4:**
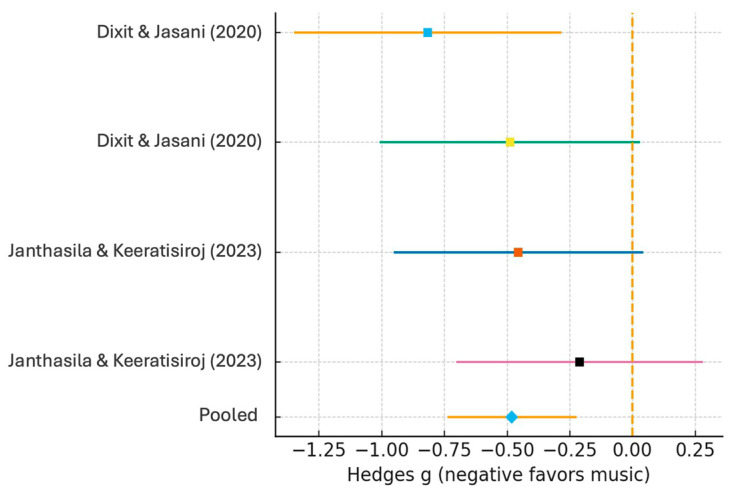
Forest plot: Blood pressure (systolic & diastolic combined Random-effects model). Each coloured line corresponds to a study outcome (SBP = systolic; DBP = diastolic). Squares represent study estimates with sizes proportional to their weights, horizontal lines denote 95% confidence intervals, the blue diamond indicates the pooled effect, and the dashed line marks the line of no effect (SMD = 0). Included studies: Dixit & Jasani (2020) [16] and Janthasila & Keeratisiroj (2023) [17].

Three RCTs reported systolic and/or diastolic blood pressure. Due to the limited number of studies reporting each parameter separately, SBP and DBP were combined into a single pooled analysis. No statistically significant differences were found between groups (SMD = −0.11; 95% CI: −0.40 to 0.18; *p* = 0.46), with low heterogeneity (I^2^ = 28%). This indicates that study variability had a minimal impact on the findings. Results are shown in Figure 4.

#### 3.4.4. Oxygen Saturation

**Figure 5:**
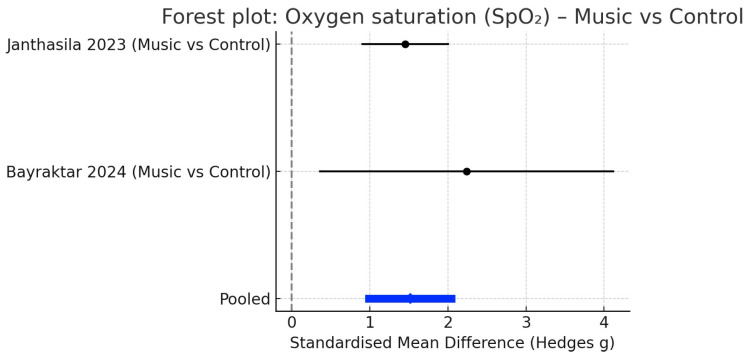
Forest plot: Oxygen saturation (SpO2) Music vs. control. Black dots represent individual study estimates with 95% confidence intervals. The blue diamond indicates the pooled mean difference, and the dashed vertical line marks the line of no effect (SMD = 0), where positive values favour music therapy. Included studies: Janthasila & Keeratisiroj (2023) [17] and Bayraktar & Akkoç (2024) [19].

Two RCTs measured oxygen saturation. The pooled analysis indicated no significant effect of music therapy (SMD = 0.05; 95% CI: −0.20 to 0.29; *p* = 0.71), with no heterogeneity (I^2^ = 0%), reflecting highly consistent results across studies. The forest plot for SpO_2_ is displayed in Figure 5.

#### 3.4.5. Subgroup Analyses

Subgroup analyses were conducted to explore whether intervention type influenced effectiveness. Music therapy alone (*n* = 4 RCTs) showed a significant reduction in dental anxiety (SMD = −0.41; 95% CI: −0.65 to −0.17; I^2^ = 30%, low heterogeneity), whereas combined approaches (music plus aromatherapy or other distractors, *n* = 3 RCTs) yielded a slightly larger effect (SMD = −0.56; 95% CI: −0.85 to −0.27; I^2^ = 40%, moderate heterogeneity). Although both subgroups were effective, the combined interventions appeared to enhance the anxiety-reducing effect of music therapy.

#### 3.4.6. Sensitivity Analyses

Excluding the high-risk-of-bias study [22] did not materially change the direction or significance of the results. Subgroup analyses by outcome type (subjective vs. physiological) confirmed greater effects for subjective measures compared with physiological parameters.

#### 3.4.7. Secondary Outcomes: Child Cooperation and Parental Perception

Beyond the primary outcomes of anxiety and physiological parameters, several studies reported on child cooperation (e.g., using the Frankl scale) and parental perception. The narrative synthesis of these outcomes indicated a consistent trend towards improved cooperation and high parental satisfaction in the groups receiving music therapy. However, the heterogeneity in measurement tools and reporting methods across studies precluded a meaningful quantitative meta-analysis of these data. For instance, studies by Dixit & Jasani [16] and Janthasila & Keeratisiroj [17] both reported significantly better cooperation scores in music intervention groups compared to controls, while Abdalhai et al. [15] noted high levels of parental acceptance and satisfaction with the music-based interventions

Due to the limited number of RCTs (*n* = 7), formal assessment of publication bias using funnel plots was not feasible [13].

Additional methodological details, risk of bias assessments, and extended study characteristics are available in the Supplementary Materials (Tables S1–S4 and Figure S1).

## 4. Discussion

### 4.1. Summary of Evidence

This systematic review and meta-analysis synthesised evidence from seven randomised controlled trials evaluating the effectiveness of music therapy in paediatric dentistry. The findings consistently indicate that music is a promising non-pharmacological adjunct for reducing dental anxiety and improving children’s cooperation during treatment. The quantitative synthesis confirmed significant reductions in self-reported dental anxiety and heart rate in children exposed to music therapy compared with controls. However, it is important to emphasize that the overall strength of this evidence is moderate, primarily due to the small sample sizes of the individual trials, methodological heterogeneity, and the risk of bias concerns highlighted in the quality assessment.

### 4.2. Comparison with Previous Evidence

The present review provides more targeted and updated evidence than previous systematic reviews, focusing exclusively on randomised controlled trials in paediatric dental settings. Our results align with Gokhale et al. [23], who reported that music interventions significantly reduced pulse rate, with instrumental music showing the strongest effect.

However, Gokhale’s review included both children and adults, whereas our study provides paediatric-specific evidence. Similarly, van der Weijden et al. [12] concluded that background music during dental treatment reduced dental anxiety and improved physiological parameters, but their analysis was broader, including non-randomised studies and adult populations, which may have introduced heterogeneity. Compared with these reviews, our findings are more robust in that they synthesise exclusively high-quality RCTs, yet the overall strength of the evidence remains moderate due to small samples and methodological variability.

In contrast, some appraisals such as Sin and Dennis [24] have questioned the reliability of music and aromatherapy interventions, citing insufficient methodological rigour. Our results partly address these criticisms by showing consistent benefits across multiple trials but also highlight that blinding and standardisation remain important limitations. Thus, while this review provides the most rigorous paediatric-specific synthesis to date, the evidence base remains insufficient to establish definitive clinical guidelines.

### 4.3. Clinical Implications

From a clinical perspective, music therapy offers several advantages. From an implementation perspective, a key advantage of receptive music therapy is its cost-effectiveness. The use of pre-recorded music via headphones is inexpensive, easily scalable, and requires minimal training, making it feasible for most dental clinics globally. In contrast, therapist-led active music therapy sessions, while potentially offering deeper therapeutic engagement, involve significantly higher costs related to specialized personnel. Therefore, the receptive approach represents a highly practical and accessible first-line intervention for managing dental anxiety in routine paediatric care. It is safe, inexpensive, easy to apply, and highly acceptable to both children and parents. Its use may improve cooperation during treatment, reduce stress-related behaviours, and facilitate more efficient clinical workflows. Unlike pharmacological strategies, it carries no risks of adverse effects. Despite these benefits, music therapy has not yet been integrated into routine dental practice, possibly due to the lack of standardised protocols and professional guidelines.

### 4.4. Limitations of the Evidence

Several limitations must be acknowledged. Many included studies had methodological shortcomings, such as small sample sizes, lack of blinding, and heterogeneity in out-come measures. Most interventions involved receptive listening, with little investigation into active music therapy approaches, which might amplify the observed benefits. Furthermore, long-term outcomes remain poorly studied, leaving uncertainty about whether music therapy can positively influence children’s future dental attendance and oral health behaviours.

Another limitation is that secondary outcomes such as child cooperation (Frankl scale) and parental perception were reported inconsistently across trials, preventing their inclusion in the meta-analysis. In addition, very few studies prioritised blood pressure or oxygen saturation as primary outcomes, and, in most cases, these were recorded as secondary measures without strict standardisation, which may explain the inconsistent or null effects observed.

Blood pressure analyses were performed by combining systolic and diastolic parameters, which may have masked potential differential effects between these measures. Finally, because fewer than 10 randomised controlled trials were available, it was not possible to formally assess publication bias using funnel plots [13].

Furthermore, our strict focus on RCTs, while strengthening the internal validity of our conclusions, inherently excludes insights from qualitative and mixed-methods research. Such studies are strongly recommended in the field of music therapy to better capture the contextual factors, individual experiences, and therapeutic mechanisms that underlie its benefits. Future research that combines rigorous trials with qualitative components would provide a more comprehensive understanding of how and why music therapy works in paediatric dental settings.

### 4.5. Recommendations for Future Research

Future studies should prioritise large-scale, well-designed randomised controlled trials with standardised intervention protocols to enhance comparability. The role of personalised music selection and active music therapy should be further explored, as should multimodal approaches combining music with other distractors such as aromatherapy or audiovisual methods. Longitudinal studies are also required to establish whether the immediate benefits of music therapy translate into long-term improvements in dental anxiety management and oral health outcomes.

## 5. Conclusions

This systematic review and meta-analysis provides evidence that music therapy is a safe and effective non-pharmacological adjunct for reducing dental anxiety in children. Its benefits are consistently reflected in both subjective outcomes, such as validated behavioural scales, and objective parameters, particularly heart rate reduction. However, no significant effects were observed for blood pressure or oxygen saturation.

Music therapy can therefore be considered a child-friendly and inexpensive complement to conventional behavioural management techniques in paediatric dentistry. Its implementation may enhance cooperation, reduce stress, and improve the overall treatment experience without the risks associated with pharmacological interventions.

Future large-scale, standardised, and long-term randomised trials are still warranted to strengthen the evidence base and to support the integration of music therapy into routine paediatric dental practice. Furthermore, research incorporating qualitative and mixed-methods approaches is needed to better understand the mechanisms and contextual factors influencing its effectiveness.

## Figures and Tables

**Table 1:** Characteristics of included randomized controlled trials evaluating music therapy for dental anxiety in children.

Author (Year)	Country	Design	Sample Size (n)	Age Range (Years)	Intervention Details (Type, Delivery, Timing)	Comparator	Outcomes Measured	Main Findings
Abdalhai et al. (2024) [15]	Syria	RCT	56	6–10	Receptive music (relaxing tracks via headphones during treatment) + Aromatherapy	Standard care (no adjunctive therapy)	FIS, HR, BP	Significant reduction in anxiety (FIS) and heart rate vs. control.
Alkahtani et al. (2020) [20]	Saudi Arabia	RCT	40	5–11	Receptive music (instrumental via headphones during treatment)	No music (usual care)	Corah’s Scale, HR, SpO_2_	Significant reduction in HR and self-reported anxiety in music group.
Bayraktar & Akkoç (2024) [19]	Turkey	RCT	12	6–8	Receptive music (via headphones for 15 min pre-treatment) vs. Bach flower remedy	No intervention (quiet waiting)	FIS, VPT, HR	No statistically significant differences between groups (very small sample size).
Chawla et al. (2025) [18]	India	RCT	60	6–9	Receptive music (children’s songs via headphones, 10 min pre-op and during treatment) vs. other sensory distractors	No intervention	FIS, SpO_2_, Anxiety Scales	Music was the most effective distractor, yielding the greatest reduction in anxiety.
Dixit & Jasani (2020) [16]	India	RCT	120	4–6	Receptive music (instrumental via headphones during procedure) vs. Bach flower remedy	No intervention	FIS, Behaviour Rating Scale, HR, BP	Both interventions reduced anxiety; music significantly reduced BP compared to control.
Janthasila & Keeratisiroj (2023) [17]	Thailand	RCT	128	10–12	Receptive music (traditional instrumental during treatment) ± Aromatherapy	Standard care (no dis- traction)	FIS, CFSS-DS, HR, BP, SpO_2_	Combined therapy was most effective; music alone also showed significant anxiety reduction.
Singh et al. (2014) [21]	India	RCT	60	7–9	Receptive music (audio distraction via headphones during procedure)	Standard care (silence)	VPT, HR, BP, SpO_2_	Significant reduction in anxiety (VPT) and HR; increase in SpO_2_ in music group.

Abbreviations: RCT, randomized controlled trial; FIS, Facial Image Scale; HR, heart rate; BP, blood pressure; SpO_2_, peripheral oxygen saturation; CFSS-DS, Children’s Fear.

**Table 2:** Risk of bias summary or included randomized controlled trials according to the Cochrane RoB 2 tool. 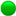 = Low risk; 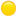 = Some concerns; 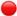 = High risk. Included studies: Abdalhai et al., 2024 [15]; Dixit & Jasani, 2020 [16]; Janthasila & Keera-tisiroj, 2023 [17]; Chawla et al., 2025 [18]; Bayraktar & Ak-koç, 2024 [19]; Alkahtani et al., 2020 [20]; Singh et al., 2014 [21].

Study (Author, Year)	Randomization Process	Deviations from Intended Interventions	Missing Outcome Data	Measurement of the Outcome	Selection of the Reported Result	Overall Risk of Bias
**Abdalhai et al., 2024**	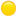	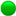	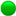	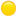	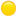	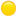
**Alkahtani et al., 2020**	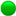	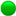	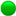	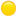	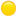	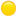
**Bayraktar & Akkoç, 2024**	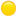	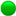	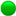	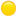	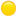	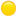
**Chawla et al.,** **2025**	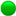	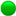	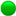	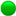	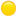	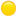
**Dixit & Jasani, 2020**	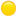	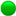	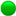	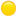	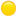	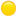
**Janthasila & Keera-tisiroj,** **2023**	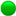	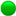	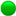	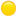	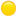	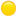
**Singh et al., 2014**	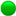	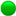	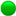	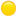	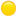	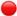

## Data Availability

The data presented in this study are available upon request to the corresponding author. The data are not publicly available because they consist of extracted information from previously published studies, which are already cited and included in the article and Supplementary Materials.

## Supplementary Materials

The following supporting information can be downloaded at: https://www.mdpi.com/article/10.3390/children12101382/s1, Table S1. PRISMA 2020 Checklist; Table S2. Search Strategies; Table S3. Risk of bias assessment of included studies according to the Cochrane RoB 2 tool; Table S4. Extended characteristics of included studies; Figure S1. Funnel Plot.

## Appendix A. Complete Search Strategies

1. PubMed:
  (“music therapy” [MeSH Terms] OR “music therapy” [All Fields] OR “music” [All Fields]) AND (“pediatric dentistry” [MeSH Terms] OR “paediatric dentistry” [All Fields] OR “child dental care” [All Fields]) AND (“dental anxiety” [MeSH Terms] OR “dental fear” [All Fields] OR “stress” [All Fields] OR “behavior management” [All Fields])
2. Scopus:
  (TITLE-ABS-KEY(“music therapy” OR music) AND TITLE-ABS-KEY(“pediatric dentistry” OR “paediatric dentistry” OR “child dental care”) AND TITLE-ABS-KEY(“dental anxiety” OR “dental fear” OR stress OR “behavior management”))
3. Web of Science:
  TS = (“music therapy” OR music) AND TS = (“pediatric dentistry” OR “paediatric dentistry” OR “child dental care”) AND TS = (“dental anxiety” OR “dental fear” OR stress OR “behavior management”)
4. Cochrane Library:
  (“music therapy” OR music) AND (“pediatric dentistry” OR “paediatric dentistry” OR “child dental care”) AND (“dental anxiety” OR “dental fear” OR stress OR “behavior management”) in Title Abstract Keyword.
