# Supplementary material for: Music Therapy for Managing Dental Anxiety in Children: A Systematic Review and Meta-Analysis of Clinical Evidence
Source: Children (Basel). 2025 Oct 13;12(10):1382. doi: 10.3390/children12101382 (PMC12563863; doi:10.3390/children12101382)
Supplement: Supplementary file 1 [file children-12-01382-s001.zip › children-3903558-supplementary.pdf]

Supplementary Material

Table S1. PRISMA 2020 Checklist

Section/Topic	Item	Checklist Item	Reported on
Title	1	Identify the report as a systematic review.	Title page
Abstract	2	See PRISMA 2020 for Abstracts checklist.	Abstract
Introduction	3	Rationale: Describe the rationale for the review in the context of what is already known.	pp. 2–3
Introduction	4	Objectives: Provide an explicit statement of the objectives or questions being addressed.	p. 3
Methods	5	Eligibility criteria: Specify inclusion and exclusion criteria for the review.	pp. 3–4
Methods	6	Information sources: Specify all databases and other sources searched or consulted.	p. 4
Methods	7	Search strategy: Present the full search strategies for all databases, registers, and websites.	Supplementary Table S2
Methods	8	Selection process: Specify methods used to decide whether a study met inclusion criteria.	p. 4
Methods	9	Data collection process: Specify methods used to collect data from reports.	pp. 4–5
Methods	10a	Data items: List and define all outcomes for which data were sought.	pp. 4–5
Methods	10b	Data items: List and define all other variables for which data were sought.	pp. 4–5
Methods	11	Risk of bias assessment: Specify methods used to assess risk of bias.	p. 5

Methods	12	Effect measures: Specify for each outcome the effect measures used.	p. 5
Methods	13a	Synthesis methods: Processes used to decide study eligibility for each synthesis.	pp. 5–6
Methods	13b	Methods to prepare the data for presentation/synthesis.	pp. 5–6
Methods	13c	Methods to tabulate or visually display results.	Tables 1–2; Figures 1–6
Methods	13d	Methods to synthesize results and rationale.	pp. 5–6
Methods	13e	Methods to explore heterogeneity.	p. 6
Methods	13f	Sensitivity analyses conducted.	p. 6
Methods	14	Reporting bias assessment: Methods to assess risk of bias due to missing results.	p. 6
Methods	15	Certainty assessment: Methods to assess certainty/confidence in evidence.	Not conducted (n=7 RCTs only)
Results	16a	Results of the search and selection process.	pp. 6–7; Fig. 1
Results	16b	Cite studies that met inclusion criteria.	pp. 6–7; Table 1
Results	17	Study characteristics.	Table 1; Supplementary Table S4
Results	18	Risk of bias in studies.	p. 7; Fig. 2; Table S3
Results	19	Results of individual studies.	pp. 7–8; Table 1
Results	20a	Results of syntheses: summary of characteristics and risk of bias.	pp. 7–8
Results	20b	Results of all statistical syntheses.	pp. 8–9; Figures 3–6
Results	20c	Results of heterogeneity investigations.	p. 9
Results	20d	Results of sensitivity analyses.	p. 9
Results	21	Reporting biases.	p. 9
Results	22	Certainty of evidence.	Not assessed
Discussion	23a	Interpretation of results.	pp. 9–11
Discussion	23b	Limitations of evidence.	pp. 11–12
Discussion	23c	Limitations of review	pp. 11–12

		process.	
Discussion	23d	Implications for practice, policy, and research.	pp. 11–12
Other Information	24a	Registration and protocol information.	p. 3 (PROSPERO CRD420251138312)
Other Information	24b	Indicate where protocol is accessible.	PROSPERO
Other Information	24c	Amendments to protocol.	None
Other Information	25	Support: sources of financial/non-financial support.	p. 12
Other Information	26	Competing interests.	p. 12
Other Information	27	Availability of data, code, materials.	p. 12

Table S2. Search Strategies

Database	Search strategy	Date of search	Results
PubMed	("music therapy"[MeSH Terms] OR "music"[All Fields]) AND ("pediatric dentistry"[MeSH Terms] OR "paediatric dentistry"[All Fields]) AND ("dental anxiety"[MeSH Terms] OR "dental fear"[All Fields])	August 2025	50
Scopus	TITLE-ABS-KEY (music AND (pediatric OR paediatric) AND (dentistry OR dental) AND (anxiety OR fear))	August 2025	23
Web of Science	TS=(music AND pediatric AND dentistry AND anxiety)	August 2025	27
Cochrane Library	(music therapy AND dental anxiety AND child*)	August 2025	0

Table S3. Risk of bias assessment of included studies according to the Cochrane RoB 2 tool

Study (Author, Year)	Randomisation process	Deviations from intended interventions	Missing outcome data	Measurement of the outcome	Selection of reported result	Overall risk of bias
Abdalhai et al., 2024	Some concerns	Low risk	Low risk	Some concerns	Some concerns	Moderate
Alkahtani et al., 2020	Low risk	Low risk	Low risk	Some concerns	Some concerns	Moderate
Bayraktar & Akkoç, 2024	Some concerns	Low risk	Low risk	Some concerns	Some concerns	Moderate
Chawla et al., 2025	Low risk	Low risk	Low risk	Low risk	Some concerns	Low–moderate
Dixit & Jasani, 2020	Some concerns	Low risk	Low risk	Some concerns	Some concerns	Moderate
Janthasila & Keeratisiroj, 2023	Low risk	Low risk	Low risk	Some concerns	Some concerns	Moderate
Singh et al., 2014	Low risk	Low risk	Low risk	Some concerns	Some concerns	Moderate

Figure S1. Funnel Plot

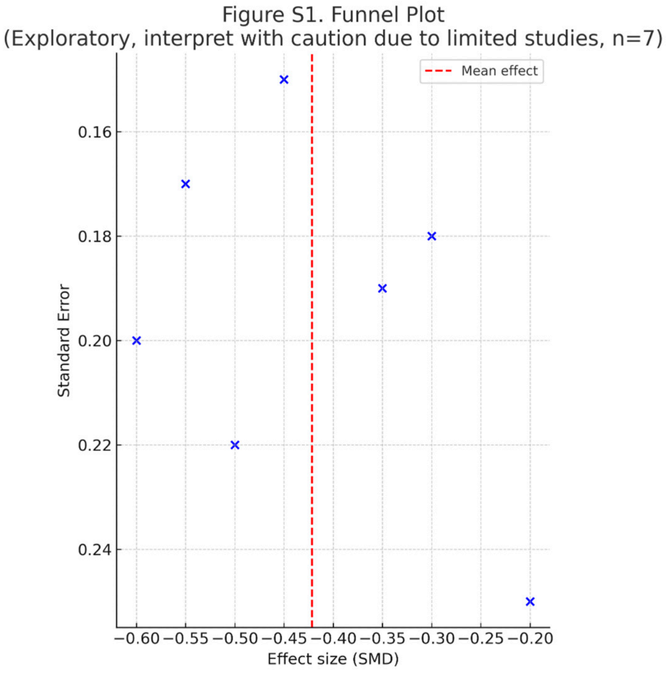

An exploratory funnel plot was generated but should be interpreted with caution due to the limited number of studies (n=7).

Table S4. Extended characteristics of included studies

Author (Year)	Country	Design	Sample size (n)	Age range (years)	Gender distribution	Type of music	Delivery mode	Duration	Timing	Setting	Comparator	Outcomes measured	Main findings
Abdalhai et al. (2024)	Syria	RCT	56	6–10	Not reported	Relaxing tracks	Headphones	During treatment	During	Dental clinic	Standard care (no adjunctive therapy)	FIS, HR, BP	Significant reduction in anxiety (FIS) and heart rate vs. control.
Alkahtani et al. (2020)	Saudi Arabia	RCT	40	5–11	Not reported	Instrumental music	Headphones	During treatment	During	Dental clinic	No music (usual care)	Corah's Scale, HR, SpO_2	Significant reduction in HR and self-reported anxiety in music group.
Bayraktar & Akkoç (2024)	Turkey	RCT	12	6–8	Not reported	Not specified	Headphones	15 min pre-treatment	Before	Dental clinic	No intervention (quiet waiting)	FIS, VPT, HR	No statistically significant differences (very small sample size).
Chawla et al. (2025)	India	RCT	60	6–9	Not reported	Children's songs	Headphones	10 min pre-op + during	Before & during	Dental clinic	No intervention	FIS, SpO_2, Anxiety Scales	Music was the most effective distractor,

													reducing anxiety.
Dixit & Jasani (2020)	India	RCT	120	4–6	Not reported	Instrumental music	Headphones	During procedure	During	Dental clinic	No intervention	FIS, Behaviour Rating Scale, HR, BP	Both interventions reduced anxiety; music significantly reduced BP vs. control.
Janthasila & Keeratisiroj (2023)	Thailand	RCT	128	10–12	Not reported	Traditional instrumental	Headphones	During treatment	During	Dental clinic	Standard care (no distraction)	FIS, CFSS-DS, HR, BP, SpO_2	Combined therapy most effective; music alone also reduced anxiety.
Singh et al. (2014)	India	RCT	60	7–9	Not reported	Not specified (audio distraction)	Headphones	During procedure	During	Dental clinic	Standard care (silence)	VPT, HR, BP, SpO_2	Significant reduction in anxiety (VPT) and HR; increase in SpO_2 in music group.

This table expands on Table 2 from the main text and includes further details on gender distribution, type of music, duration, timing, and setting.
